# Emergency department crowding in The Netherlands: managers’ experiences

**DOI:** 10.1186/1865-1380-6-41

**Published:** 2013-10-24

**Authors:** Christien van der Linden, Resi Reijnen, Robert W Derlet, Robert Lindeboom, Naomi van der Linden, Cees Lucas, John R Richards

**Affiliations:** 1 Accident and Emergency Department, Medical Center Haaglanden, P.O. Box 432, The Hague 2501, CK, The Netherlands; 2 Department of Emergency Medicine, PSSB 2100, 4150 V Street, Sacramento 95817, CA, USA; 3 Division of Clinical Methods and Public Health, Master Evidence Based Practice, Academic Medical Center and University of Amsterdam, P.O. Box 22660, Amsterdam 1100, DD, The Netherlands; 4 Institute for Medical Technology Assessment, Erasmus University Rotterdam, P.O. Box 1738, Rotterdam 3000, DR, The Netherlands

**Keywords:** Emergency service, Hospital, Emergency department crowding, Overcrowding

## Abstract

**Background:**

In The Netherlands, the state of emergency department (ED) crowding is unknown. Anecdotal evidence suggests that current ED patients experience a longer length of stay (LOS) compared to some years ago, which is indicative of ED crowding. However, no multicenter studies have been performed to quantify LOS and assess crowding at Dutch EDs. We performed this study to describe the current state of emergency departments in The Netherlands regarding patients’ length of stay and ED nurse managers’ experiences of crowding.

**Methods:**

A survey was sent to all 94 ED nurse managers in The Netherlands with questions regarding the type of facility, annual ED census, and patients’ LOS. Additional questions included whether crowding was ever a problem at the particular ED, how often it occurred, which time periods had the worst episodes of crowding, and what measures the particular ED had undertaken to improve patient flow.

**Results:**

Surveys were collected from 63 EDs (67%). Mean annual ED visits were 24,936 (SD ± 9,840); mean LOS for discharged patients was 119 (SD ± 40) min and mean LOS for admitted patients 146 (SD ± 49) min. Consultation delays, laboratory and radiology delays, and hospital bed shortages for patients needing admission were the most cited reasons for crowding. Admitted patients had a longer LOS because of delays in obtaining inpatient beds. Thirty-nine of 57 respondents (68%) reported that crowding occurred several times a week or even daily, mostly between 12:00 and 20:00. Measures taken by hospitals to manage crowding included placing patients in hallways and using fasttrack with treatment of patients by trained nurse practitioners.

**Conclusions:**

Despite a relatively short LOS, frequent crowding appears to be a nationwide problem according to Dutch ED nurse managers, with 68% of them reporting that crowding occurred several times a week or even daily. Consultations delays, laboratory and radiology delays, and hospital bed shortage for patients needing admission were believed to be the most important factors contributing to ED crowding.

## Background

In The Netherlands, major changes in the organization of emergency care are planned to decrease health care costs. For example, the closure of 40% of the emergency departments (EDs) was recently discussed [1]. This could impact care in EDs by causing crowding. Dutch health policy makers and insurance companies plan to integrate general practitioner (GP) cooperatives and EDs into one facility to prevent patients from self-referring to the ED. Both changes may affect ED patients’ length of stay (LOS) and crowding.

Although ED crowding is not yet a major problem in this country according to expert opinion [2], anecdotal evidence suggests that current ED patients experience a longer LOS compared to some years ago, which is indicative of ED crowding. However, no multicenter studies have been performed to quantify LOS and assess crowding at Dutch EDs. We conducted this study to describe the current state of EDs in The Netherlands, including ED characteristics, patients’ LOS, and ED nurse managers’ experiences of crowding. To study the effect of the planned changes in the organization of emergency care on ED patients’ LOS and ED crowding, we plan to repeat this study in 3 years.

## Methods

### Study setting and study design

In The Netherlands, there are 132 hospital locations. Ninety-nine hospital locations have EDs [3], including 91 general hospitals and 8 university hospitals. There are an estimated 2.2 million ED visits annually [4]. Basic health insurance is available to all citizens: half of health care is paid by taxes and employers, half by insurance. Most people are registered with a local GP. The presence of emergency physicians (EPs) is increasing [5]. To date, there are almost 300 trained and registered EPs working in 80% of the EDs [6].

A survey study in The Netherlands was performed in November 2012. The survey was addressed to the ED management; it could be completed by a nurse manager, staff nurse, medical manager, or EP. Surveys were returned to the primary investigator. Data were entered into SPSS 20.0 (SPSS Inc., Chicago, IL, USA). Institutional review board exemption was granted.

### Study protocol

At the onset of this study, a letter announcing the survey was published on the website of the Dutch Association of ED Nurses (NVSHV) and was also noted by the national press. Surveys were distributed to all ED nurse managers using an address list published by the Ministry of Health [7] combined with an address list obtained from the NVSHV. Included in the e-mail were: a letter explaining the survey, its purpose, and a digital version of the survey. A paper-based version of the survey and a second e-mailing with an online version of the survey were sent to non-respondent EDs in January 2013 to increase the response rate.

### Survey content and definitions

A draft survey was created, and after consultation of experts (two EPs and two ED nurse managers), a final version was provided (Appendix).

The survey included questions regarding type of facility, hospital size, annual ED census (based on year 2011), change in volume of annual ED visits from 2008 to 2012, ED bed capacity, number of ED nurses and physicians per shift, patients’ LOS, percentage of self-referred patients (self-referrals), percentage of patients arriving by ambulance, admission rates, and how often ambulance diversion was used. Additional questions included how often crowding occurred, which time periods had the worst episodes of crowding, putative causes of crowding, and what measures had been undertaken to improve patient flow. Respondents chose from a list of causes of crowding and from a list of measures to manage crowding. Respondents were instructed to circle all appropriate answers, creating the possibility of more than one answer per respondent. Respondents were also provided the opportunity to fill in answers other than the answer lists provided. If actual data from hospital databases were not available, respondents were allowed to report estimations. They were also allowed to skip questions.

LOS was defined as the interval between patient registration and the moment the patient left the ED. Based on previous research, crowding was defined as having more patients in the ED than treatment rooms or more patients than staff should ideally care for [8], and overcrowding was defined as dangerously crowded, with an extreme volume of patients in ED treatment areas forcing the ED to operate beyond its capacity [9].

### Data analysis

Data were reported as mean and standard deviation and median and ranges, in case of a skewed distribution. To investigate whether differences occurred by type of hospital, we examined the data for the overall group as well as for type of facility separately, using two-tailed *t* tests, Kruskal-Wallis test for ordered categories and Fisher’s exact tests where appropriate. Statistical significance was assumed at a level of *p* ≤ 0.05.

## Results

Surveys were collected from 63 EDs (64%); 36 surveys were received after the initial call, and 27 surveys were received after the reminder mail. There were 55 general and 8 university hospitals, which accounted for 56% of total general hospitals and 100% of total university hospitals participating. Respondents were ED nurse managers (*n* = 62, 98%) and one ED nurse. Six ED nurse managers were assisted by an EP, ED nurse or staff advisor. Not all respondents answered every question. The total number responding to each question is reported throughout the results and tables.

### Emergency department characteristics

Mean number of annual ED visits (± SD) in 2011 was 24,936 (*n* = 61). Mean number of annual ED visits to general hospitals (*n* = 53; 24,601) was not statistically different from mean number of annual ED visits to university hospitals (*n* = 8; 27,155) (Table 1). Fifty-six respondents (89%) answered the question about change in volume of annual ED visits from 2008 to 2012. Forty-three of them (77%) reported an increase in ED visits between 2008 and 2011, while 13 (23%) reported a decrease.

**Table 1:** **Emergency department characteristics (*n*** **= 63)**

	**Mean (SD)**	**Median**	**Range**	**Responding hospitals, *n *** **(%)**
Annual ED visits	24,936 (9,840)	24,000	7,972-52,400	61 (97)
General hospitals	24,601 (10,331)	23,625	7,972-52,400	53 (84)
University hospitals	27,155 (5,535)	26,903	19,487-34,500	8 (100)
No. of ED beds	16 (6)	16	4-28	60 (95)
No. of ED nurses				
Day shift	4.48 (1.56)	4	2-10	60 (95)
Evening shift	4.78 (1.89)	4.5	2-12	60 (95)
Night shift	2.82 (0.97)	3	1-6	60 (95)
No. of physicians				
Day shift	5.71 (3.23)	5	1-14	57 (90)
Evening shift	4.90 (2.94)	4	1-12	57 (90)
Night shift	3.41 (2.47)	2	1-10	56 (89)
Percentage of ED visits arriving by ambulance	17 (9)	15	5-60	55 (87)
Percentage of ED visits by self-referrals	35 (19)	33	3-71	58 (92)
No. of staffed beds in hospital	486 (287)	365	140-1,300	52 (83)
No. of ICU beds in hospital	16 (16)	12	3-88	51 (81)
No. of ED patients admitted for inpatient care	7,606 (2,653)	7,267	3,367-13,290	24 (38)
Percentage of ED patients admitted for inpatient care	32 (10)	33	15-55	33 (52)
ED LOS undifferentiated, min	131 (21)	135	90-163	11 (18)
ED LOS discharged patients, min	119 (40)	118	45-220	39 (62)
ED LOS for admitted patients, min	146 (49)	150	15-217	37 (59)
**Change in volume of annual ED visits from 2008 to 2012**				
**Increased ED visits**^**1**^	1,634 (1,589)	1,042	59-6,477	43 (68)
General hospitals	1,541 (1,469)	1,016	59-5,283	37 (67)
University hospitals	2,206 (2,280)	1,359	500-6,477	6 (75)
**Decreased visits**	2,405 (1,761)	1,566	738-6,376	13 (21)
General hospitals	2,427 (1,902)	1,500	738-6,376	11 (20)
University hospitals	2,281 (1,010)	2,280	1,566-2,995	2 (25)

^1^Estimations and actual data.

The characteristics of the EDs differed greatly. The mean percentage of ED patients arriving per ambulance (55 respondents) was 17%, varying from 5% to 60%; the mean percentage of self-referrals (58 respondents) was 35%, varying from 3% to 71%; and the mean percentage of ED patients admitted for inpatient care (33 respondents) was 32%, varying from 15% to 55% (Table 1).

### Length of stay

Mean LOS for discharged patients was 119 min. Mean LOS for admitted patients was 146 min. Eleven respondents estimated undifferentiated LOS only, with a mean LOS of 131 min (Table 1). The LOS in university hospitals was not significantly different from the LOS in general hospitals (discharged patients: 140 vs. 117 min, *p* = 0.27; admitted patients: 177 vs. 144 min, *p* = 0.27).

### Respondents’ experiences of crowding

Thirty-nine of the 57 respondents (68%) reported that crowding occurred two or more times a week (Table 2). No difference was found in crowding between university and general hospitals. The EDs who reported crowding also reported overcrowding (two or more times a week) in 19 cases (49%) (Table 3). University hospitals suffered from overcrowding significantly more. Sixty percent of the respondents indicated crowding occurred mostly between 12:00 and 20:00. Respondents mentioned consultation delays (*n* = 51, 80%) most frequently as a problem contributing to crowding, and radiology and laboratory delays (*n* = 44, 70%) also ranked highly (Table 4). Patients referred to the ED by GPs were considered to contribute most to crowding, followed by multi-trauma patients (Table 5).

**Table 2:** **EDs reporting crowding, by annual ED volume and type of facility (*n*** **= 57)**

	Crowding*, *n * **(%)**	**No crowding, *n *** **(%)**	***P***
Annual ED volume	39 (68)	18 (32)	0.64^1^
>40,000 visits	4	1	
30,001-40,000 visits	9	2	
20,000-30,000 visits	16	9	
<20,000 visits	10	6	
Type of hospital			1.0^2^
General hospital (*n* = 50)	34	16	
University hospital (*n* = 7)	5	2	

*Crowding daily or more than twice a week.

^1^Kruskal-Wallis test for ordered categories.

^2^Fisher’s exact test.

**Table 3:** **EDs reporting crowding AND overcrowding, by annual ED volume and type of facility (*n*** **= 39)**

	Crowding and overcrowding*, *n * **(%)**	**No overcrowding, *n *** **(%)**	***P***
Annual ED volume*	19 (49)	20 (51)	0.55^1^
>40,000 visits	3	1	
30,001-40,000 visits	4	5	
20,000-30,000 visits	7	9	
<20,000 visits	5	5	
Facility type			0.03^2^
General hospital	14	20	
University hospital	5	0	

*Overcrowding daily or more than twice a week.

^1^Kruskal-Wallis test for ordered categories.

^2^Fisher’s exact test.

**Table 4:** **Problems related to crowding according to the respondents (*n*** **= 63)**

**Problem**	***n *** **(%)**
Consultation delays	51 (81)
Radiology and laboratory delays	44 (70)
Delays for admitted patients/hospital bed shortage	40 (64)
Physician staff shortage	30 (48)
Insufficient ED space	29 (46)
Delays in transfer	21 (33)
Long waits in triage	20 (32)
Nursing staff shortage	15 (24)
Registration delays	3 (5)

**Table 5:** **Patients with most impact on crowding according to the respondents (*n*** **= 63)**

**Patients**	***n *** (%)*
Patients referred by a general practitioner, needing admission	27 (43)
Multi trauma patients	21 (33)
Patients admitted to an inpatient unit	18 (29)
Psychiatric patients	17 (27)
Self-referrals	10 (16)
Geriatric patients	10 (16)
Children	6 (10)

Measures to manage crowding mentioned most frequently included placing patients in hallways (*n* = 25, 40%) and implementing fast-track units for patients with minor injuries (*n* = 24, 38%) (Table 6). Ambulance diversion policies ranged from having diversion plans to a policy of never diverting patients. Twenty-two of 59 respondents (37%) claimed they never used ambulance diversion. Ambulance diversion of one to six times per year was most common, reported by 24 of the 59 responding institutions (41%) (Table 7).

**Table 6:** **Measures for handling periods of crowding (*n*** **= 63)**

**Measures**	***n (*%)**
Treating patients in non-treatment areas	25 (40)
Fast-track for minor injuries	24 (38)
Expansion of emergency physician, nursing, and ancillary staff	24 (38)
Expanding inpatient hospital bed capabilities and development of ED observational units	22 (35)
Ambulance diversion	19 (30)
Adapting the number of patients per room	16 (25)
Performing consults outside the ED area	15 (24)
Rebuilding (parts of) the ED	15 (24)
Double triage coverage	12 (19)
Implementation of a GP cooperative at the ED	12 (19)
Hiring nurse practitioners or physician assistants	10 (16)
Triaging patients out of the ED to the GP or outpatient clinic	9 (14)

**Table 7:** **Number of times EDs were on ambulance diversion (*n*** **= 59)**

	***n *** **(%)**
Never	22 (37)
1-6 times per year	24 (41)
7-12 times per year	6 (10)
2-4 times per month	6 (10)
Several times per week	1 (2)

## Discussion

LOS at EDs in The Netherlands (119 min for discharged patients, 146 min for admitted patients) is short compared to published LOSs in other countries [10,11]. In the USA, admitted patients may have an LOS of over 24 h during times of severe crowding [12]. Despite this relatively short LOS, frequent crowding appears to be a Dutch problem according to our respondents, with 68% of them reporting that crowding occurred several times a week or even daily, and half of those reporting that, besides crowding, their ED was also overcrowded two or more times a week. Our findings are somewhat milder compared to studies performed in the USA more than 10 years ago by the co-authors [13-15] in which 91% of the ED directors in the USA reported crowding to be a problem, probably indicating that crowding is better controlled in The Netherlands. However, if health restructures continue (closure of EDs and decreasing inpatient bed capacity), crowding may become more prevalent. Our respondents named several factors they believed to contribute to ED crowding, and their answers were similar to those from other international studies [16-19]: consultation delays, shortages in ED space and beds, admission delays, shortages of acute care inpatient beds, lack of nursing staff, and laboratory and radiology delays.

In the Dutch lay press, it is suggested that the problem of crowded EDs is predominantly caused by inappropriate use of emergency services by patients seeking care for non-urgent problems. The same was suggested in the USA in the early 1990s in several position statements [20]. Integration of GP with ED services has had mixed effects: unsuccessful in some hospitals in Australia and New Zealand, while effective in diverting patients in one study from The Netherlands [21,22]. This Dutch study did not measure effects on crowding. Current research on ED crowding suggests that discouraging the use of the ED for non-emergency issues will not solve the problem. Rather, the issue of output, for example, inadequate inpatient capacity for a patient population with an increasing complexity and severity of illness, is now believed to be the single most important factor contributing to ED crowding [23]. Our respondents agreed: 64% cite hospital bed shortages as a problem contributing to crowding. Only 16% blamed self-referrals for crowding, while many (43%) believed crowding occurs when too many patients who are referred by the GP or multi-trauma patients present at the ED (33%). High patient acuity has been cited as a significant contributing factor to ED crowding [13].

Besides GP cooperatives, numerous measures have been implemented to improve ED efficiency and alleviate crowding in Dutch EDs. These measures have been mentioned in the past international literature about ED crowding. Examples include implementing observation units [24] and creating a fast-track unit [25]. A few measures described in the international literature were rarely mentioned in our study, such as ambulance diversion. For many Dutch EDs, ambulance diversion is not an option, even when conditions warrant diversion. Most university and major EDs have no alternative treatment site, since EDs in The Netherlands have special assignments, such as a dedicated trauma center designation. For non-trauma ambulance patients, diversion would be possible; however, hospitals have strong economic pressures to remain open. Only one respondent reported requiring diversion several times per week.

The body of evidence documenting the adverse effects of crowding has grown up to the sky. Crowding not only compromises the quality of care, it also worsens clinical outcomes [26] and has negative effects on staff satisfaction and health [27]. It is apparent that most countries have been struggling with ED crowding for many years, and the focus has shifted from identifying causes and consequences to finding solutions. The Dutch are following this trend. Some Dutch EDs have implemented fasttrack (38%) with or without nurse practitioners (16% of the respondents use nurse practitioners), which has been reported to help decrease LOS [28]. ED nurse managers recognize that the cause and solution to ED overcrowding lie outside the ED. They consider ED crowding as a system-wide problem instead of an ED phenomenon, as seen in other countries. Facilities are increasingly utilizing ED-managed overflow units (acute admission units, transit lounges and flexible beds) to make room for incoming patients. These overflow units mitigate crowding by giving the ED staff a way to control patient outflow to some extent [29,30]. Other important potential solutions, such as expediting discharge from the main wards, were not mentioned by our ED nurse managers.

Future studies in The Netherlands should focus on determining which aspects of restructuring healthcare are most closely related to ED crowding. The Dutch can learn from what is already known in other countries with severe crowding. Despite environmental, demographic and healthcare organization differences among countries, the causes and consequences of crowding appear to be universal, and certain strategies will alleviate crowding wherever they are implemented. From the existing evidence, it is clear that multidisciplinary system-wide support is necessary to solve ED crowding. Introducing quality benchmarks in The Netherlands would be useful. Moreover, EDs should start collecting a uniform set of process measures that provides real-time observation of the operation of the department like the crowding measures recently identified by Beniuk et al. [31]. This would facilitate across-facility comparisons to identify the best practices that work in our healthcare system.

### Limitations

First, our survey has not been validated yet. As in most surveys, our results are subject to reporting errors, non-response, and incomplete responses. In The Netherlands, several different patient information systems are used, and hospitals use different definitions for the data that are tracked. For example, referral source and transport were used interchangeably at different sites: in some EDs, all patients brought in by ambulance were documented as 'ambulancepatients’, while in other EDs patients who were referred by a GP but transported by an ambulance were not registered as such. In some Dutch EDs, visits are not tracked, so a few respondents presented estimations instead of actual data. Although this data collection is far from ideal, we believe the benefits of multicenter participation outweighed the weaknesses of variation in operational data. We do not know if the characteristics of non-responding EDs were similar or systematically different from those of responding EDs. However, our purpose was not to assess the population as a whole but rather to describe the current status of EDs, current LOS, and ED nurse managers’ experiences of crowding.

Another major limitation is that no standard definition of ED crowding exists [16,32]. Several factors associated with crowding were included in the survey, but no standard method was used for actually defining crowding. ED crowding assessment tools (e.g., EDWIN [33], NEDOCS [34]) are not yet used routinely in The Netherlands. Some metrics that define patient throughput, such as ambulance diversion hours [35] or the number of patients leaving without being seen [36], are used as surrogate markers of crowding in the absence of a widely accepted definition [37]. Measuring crowding with hours on ambulance diversion or with the percentage of patients leaving without being seen will not give a true picture of ED conditions in The Netherlands, since both circumstances are rare. As with other studies [33,38], we used staff perceptions of crowding. Although subjective, ED nurse managers’ sense of how their EDs operate was the closest accurate measure of current crowding. After national implementation of crowding measures into the ED information system in The Netherlands, further studies assessing ED crowding will be necessary, using empirical data to quantify ED nurse managers’ experiences.

## Conclusions

Despite a relatively short LOS, frequent crowding appears to be a nationwide problem according to Dutch ED nurse managers, with 68% of them reporting that crowding occurred several times a week or even daily. Almost half of the crowded EDs experienced overcrowding two or more times a week. Delays in consultations and laboratory and radiology services contributed to the problem. Admitted patients had a longer LOS because of delays in obtaining inpatient beds.

## Appendix

### The 2012 emergency department survey

Questions used for the article “Emergency Departments in The Netherlands: managers' experiences” by Christien van der Linden, Resi Reijnen, Robert W. Derlet, Robert Lindeboom, Naomi van der Linden, Cees Lucas, and John R. Richards.

1. Name and location of the hospital.

2. Function of the applicant (ED nurse manager; ED nurse; EP; other).

3. Type of facility (general or university hospital).

4. Number of staffed beds in hospital.

5. Number of ICU beds in hospital.

6. Annual ED visits in 2008.

7. Annual ED visits in 2011.

8. Number of ED beds.

9. Number of ED nurses and physicians per shift.

10. Patients length of stay (LOS), undifferentiated.

11. LOS for treat-and-release patients.

12. LOS for admitted patients.

13. Number and/or percentage of ED visits by self-referred patients.

14. Number and/or percentage of ED patients arriving by ambulance.

15. Number and/or percentage of patients admitted.

16. How often does crowding occur? (Never; 1–6 times per year; 7 to 12 times per year, 2 to 4 times per month; several times per week; daily).

17. How often does overcrowding occur? (Never; 1–6 times per year; 7 to 12 times per year; 2 to 4 times per month; several times per week; daily).

18. Which time period has the worst episodes of crowding? (24–4 h; 4–8 h; 8–12 h; 12–16 h; 16–20 h; 20–24 h).

19. Causes of crowding (consultation delays; radiology and laboratory delays; delays for admitted patients/hospital bed shortage; physician staff shortage; insufficient ED space; delays in transfer; long waits in triage; nursing staff shortage; registration delays; other).

20. Which patients have the most impact on crowding? (patients referred by a general practitioner, needing admission; multitrauma patients; patients admitted to an inpatient unit; psychiatric patients; self-referred patients; geriatric patients; children; other).

21. Measures to manage crowding (treating patients in non-treatment areas; fasttrack for minor injuries; expansion of EP, nursing, and ancillary staff; expanding inpatient hospital bed capabilities and development of ED observational units; ambulance diversion; adapting the number of patients per room; performing consultations outside the ED area; rebuilding (parts of) the ED; double triage coverage; implementation of a GP cooperative at the ED; hiring nurse practitioners or physician assistants; triaging patients out of the ED to the GP or outpatient clinic; other).

22. How often ambulance diversion is used (Never; 1–6 times per year; 7 to 12 times per year; 2 to 4 times per month; several times per week; daily).

## Abbreviations

ED: Emergency department; EP: Emergency physician; GP: General practitioner; ICU: Intensive care unit; LOS: Length of stay; NVSHV: Dutch Association of ED Nurses.

## Competing interests

The authors declare that they have no competing interests.

## Authors’ contributions

CvdL had full access to all of the data in the study and takes responsibility for the integrity of the data and the accuracy of the data analysis. Study concept and design: CvdL, RR, NvdL. Acquisition of the data: CvdL, RR. Analysis and interpretation of data: CvdL, RR, NvdL. Drafting of the manuscript: CvdL, RR, RD, RL, JR. Critical revision of the manuscript for important intellectual content: RD, RL, CL, JR. All authors read and approved the final manuscript.
